# Molecular characterization and the effect of salinity on cyanobacterial diversity in the rice fields of Eastern Uttar Pradesh, India

**DOI:** 10.1186/1746-1448-5-4

**Published:** 2009-04-06

**Authors:** Ashish Kumar Srivastava, Poonam Bhargava, Arvind Kumar, Lal Chand Rai, Brett A Neilan

**Affiliations:** 1 Department of Botany, School of Life Sciences, Mizoram University, Tanhril Campus, Aizawl-796009, India; 2 Molecular Biology Section, Center of Advanced Study in Botany, Banaras Hindu University, Varanasi-221005, India; 3 School of Biotechnology and Biomolecular Science, The University of New South Wales, Sydney, NSW 2052, Australia

## Abstract

**Background:**

Salinity is known to affect almost half of the world's irrigated lands, especially rice fields. Furthermore, cyanobacteria, one of the critical inhabitants of rice fields have been characterized at molecular level from many different geographical locations. This study, for the first time, has examined the molecular diversity of cyanobacteria inhabiting Indian rice fields which experience various levels of salinity.

**Results:**

Ten physicochemical parameters were analyzed for samples collected from twenty experimental sites. Electrical conductivity data were used to classify the soils and to investigate relationship between soil salinity and cyanobacterial diversity. The cyanobacterial communities were analyzed using semi-nested *16S rRNA *gene PCR and denaturing gradient gel electrophoresis. Out of 51 DGGE bands selected for sequencing only 31 which showed difference in sequences were subjected to further analysis. BLAST analysis revealed highest similarity for twenty nine of the sequences with cyanobacteria, and the other two to plant plastids. Clusters obtained based on morphological and molecular attributes of cyanobacteria were correlated to soil salinity. Among six different clades, clades 1, 2, 4 and 6 contained cyanobacteria inhabiting normal or low saline (having EC < 4.0 ds m^-1^) to (high) saline soils (having EC > 4.0 ds m^-1^), however, clade 5 represented the cyanobacteria inhabiting only saline soils. Whilst, clade 3 contained cyanobacteria from normal soils. The presence of DGGE band corresponding to *Aulosira *strains were present in large number of soil indicating its wide distribution over a range of salinities, as were *Nostoc*, *Anabaena*, and *Hapalosiphon *although to a lesser extent in the sites studied.

**Conclusion:**

Low salinity favored the presence of heterocystous cyanobacteria, while very high salinity mainly supported the growth of non-heterocystous genera. High nitrogen content in the low salt soils is proposed to be a result of reduced ammonia volatilization compared to the high salt soils. Although many environmental factors could potentially determine the microbial community present in these multidimensional ecosystems, changes in the diversity of cyanobacteria in rice fields was correlated to salinity.

## Background

The Indian agriculture is suffering with many man-made problems like canal irrigation, pesticide and chemical fertilization application. However, the former is responsible for salt accumulation in the soil which is further expanding due to water-logging in paddy fields. Salinization is predicted to result in 30% of farmable land loss globally within the next 25 years, and up to 50% by the year 2050 [1]. In developing countries like India and China, the problem could be more serious due to the increasing demand for rice as a staple food. If water-logged conditions prevail for lengthy durations salinization of the soil occurs and, in India, this is commonly known as the formation of Usar land [2]. High salt concentrations lead to a decline in soil fertility by adversely affecting the soil microbial flora, including nitrogen-fixing cyanobacteria and therefore further decreasing rice productivity.

Cyanobacteria, the ancient oxygen-evolving photoautotrophs, are the dominant microbial inhabitants of rice fields. Members of the orders Nostocales and Stigonematales assume a special significance in this environment [3]. Salinity adversely affects photosynthesis and therefore productivity [4], the functioning of plasma membranes [5], ionic balance in the cells [6] and protein profiles [7,8] of some phototrophs including cyanobacteria. However, salinity does not affect all cyanobacteria to the same extent due to their morphological and genomic diversity [9,10], and therefore the distribution of cyanobacterial communities in natural habitats is not uniform. The adaptive ability of cyanobacteria to salinity makes them the subject of intense biochemical and ecological investigation [11].

The classical methods for cyanobacterial identification and community assessment involve microscopic examination [3,12,13]. This assessment has, however, been criticized on the grounds that morphology can vary considerably in response to fluctuations in environmental conditions [14]. In addition, the perennating bodies of cyanobacteria such as hormogonia, akinetes and heterocysts may be difficult to characterize by microscopy and thus the actual diversity can be underestimated [15]. In view of the above, cyanobacterial diversity assessments and community analysis should be investigated by microscopic observation supplemented with a molecular taxonomy. Therefore, cyanobacterial diversity assessments using molecular tools have been widely applied [16]. The application of denaturing gradient gel electrophoresis (DGGE) along with PCR for studying natural cyanobacterial assemblages has increased our understanding of their complexity in environmental samples [17]. Among the various gene sequences used to assess cyanobacterial biodiversity, *16S rRNA *gene has been applied most often [16].

Cyanobacterial diversity has been assessed from a variety of geographical locations, including the Colorado plateau [18,19], exposed dolomite in central Switzerland [20], hot springs [21], the McMurdo Ice Self [22], and Southern Baltic Sea [23] using a combination of *16S rRNA *gene PCR and DGGE. A considerable number of studies have been done on DGGE based identification and phylogenetic characterization of toxic cyanobacteria [24-26]. In contrast to above, cyanobacteria have been characterized only at morphological level in rice fields of India [27,28], Bangladesh [29], Chile [30], Pakistan [31], Korea [32] and Uruguay [33]. However, the work of Song et al. [34] constitutes the only known report on the biodiversity assessment of cyanobacteria in rice paddy fields (Fujian, China) during September 2001 to January 2002 using molecular tools.

Despite the considerable negative impact of salinity on physiology of pure cultured cyanobacterium as observed under laboratory conditions, nothing is known regarding its effect on the biodiversity of cyanobacteria in rice fields having different salt levels. Thus there is a need to examine how salinity-induced changes among other physicochemical properties of soil affect the distribution of cyanobacteria in paddy fields. In view of the reports by Stal [35,10] that cyanobacteria have a remarkable yet varying flexibility to adapt to a wide range of environmental conditions, we propose that the resilient physiologies of certain cyanobacteria, including exopolysaccharide production, afford resistance to higher salinity compared to strains with relatively simpler morphologies. Further, high salinity inhibits ammonia volatilization [36], and this would result in soils with high nitrogen content and favor the proliferation of non-heterocystous cyanobacterial genera. This study was undertaken to provide first hand data on cyanobacterial diversity using PCR-DGGE, and correlate it to different salt levels of soil to investigate salinity-induced changes in the distribution of cyanobacteria in Indian rice fields. Further, how far the salinity affects the agriculturally important cyanobacteria was also examined.

## Results and discussion

### Physicochemical analyses of soil

**Figure 1:**
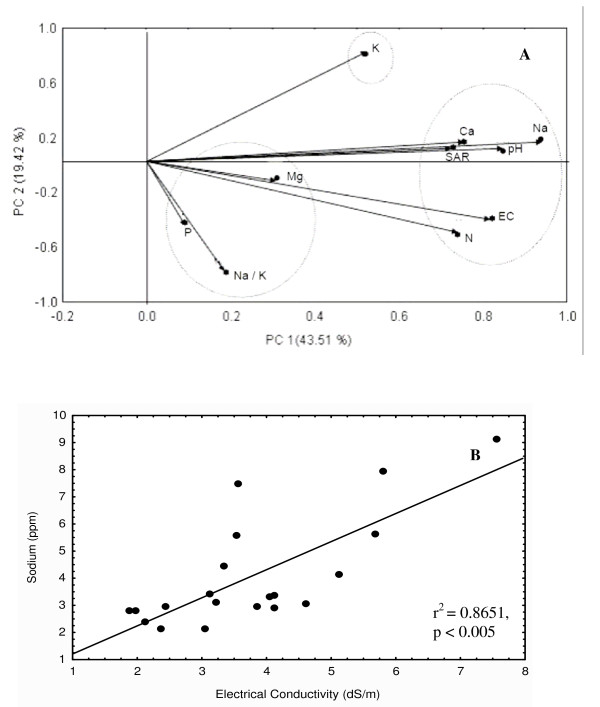
**Statistical analysis of the data of soil samples**. (A) The principal component analysis of the physicochemical properties of soil, and (B) the regression analysis between Na^+ ^concentration and electrical conductivity showing distribution of experimental sites across the regression line.

Eight different parameters, Na^+^, K^+^, Ca^2+^, Mg^2+^, Na^+^/K^+ ^ratio, SAR, EC and pH were taken into consideration for the measurement of salinity levels in soil. In addition to this, available phosphorus and total nitrogen were also estimated to determine the nutritional status of the soil. PCA analysis was performed to correlate the soil properties, especially those related to salinity, with the cyanobacterial diversity. Soil properties change significantly due to salinity in rice fields, which can ultimately determine biodiversity and hence productivity. The PCA analysis revealed two principal components (PC1 and PC2) with percentage variances of 43.51 and 19.42, respectively. The above-mentioned ten parameters distributed into three clusters (Figure 1A): (i) phosphorus, Na^+^/K^+ ^ratio and Mg^2+^, (ii) K^+ ^alone, and (iii) the remaining six parameters. This suggests that there are three major physicochemical variables that could significantly affect the cyanobacterial diversity in these rice fields. Among the different cations examined, Na^+^, which constituted the largest fraction of both soluble and exchangeable ions in the soil, had the most obvious influence on cyanobacterial distribution (indicated by the longest distance from the point of origin in the PCA plot) [37]. This result is reflected in the observation of Onkware [38] who observed deleterious effects of soil salinity (mainly Na^+^) on plant diversity and distribution in the Loburu delta of Kenya.

The sampling sites showed a wide range of Na^+ ^concentrations (2.12 – 9.15 ppm) and EC (1.89 – 7.55 ds m^-1^), thereby indicating a saline-sodic nature of the soils [39]. However, the highest EC (7.55 ds m^-1^) and Na^+ ^(9.15 ppm) were observed in the soil of Rauri. In contrast to this, the soils of Madhopur and Parasurampur had the lowest EC (1.89 ds m^-1^) and Na^+ ^(2.12 ppm) levels, respectively. The regression analysis between Na^+ ^and EC (*P *< 0.05) also showed a wide distribution of soil samples (Figure 1B). Further, K^+ ^content in saline soils was very low; lowest in the Rajatalab soil samples. This probably contributes to high Na^+^/K^+ ^and thus the sparse population of cyanobacteria observed since K^+ ^is essential for maintenance of cellular homeostasis, cell turgor and protein synthesis [40]. K^+ ^also plays a vital role in extreme environments, both as an extracellular signal and as an intracellular metabolic regulator [40] essential for growth and metabolism. Microscopically, the lower Na^+^/K^+ ^ratio was shown to support luxuriant growth of cyanobacterial mats. Although regression analysis revealed that cyanobacterial diversity decreased with an increase in Na^+^/K^+ ^ratio, a significant correlation between the number of cyanobacterial phylotypes (in terms of DGGE bands) and Na^+^/K^+ ^ratio was not confirmed. This result is in contrast to that reported by Parker et al. [41] who demonstrated K^+ ^toxicity to *Microcystis *in natural ponds. The soil of Makara also had a low Na^+^/K^+ ^ratio but was associated with a sparse cyanobacterial population, however, this could be attributed to a high pH in this case.

**Figure 2:**
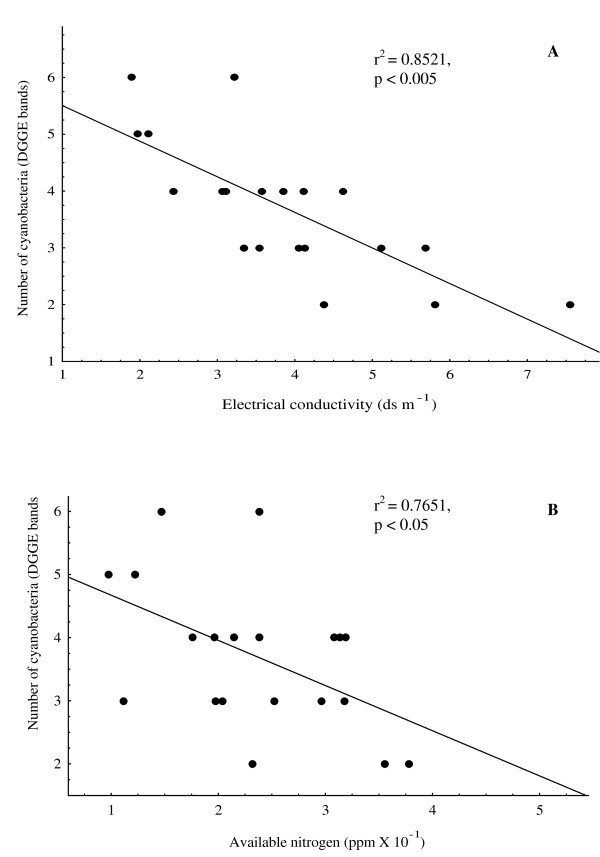
**Regression analysis between number of cyanobacteria (DGGE bands) and (A) electrical conductivity, (B) total nitrogen**. Analyses depict the effects of these parameters on cyanobacterial abundance in selected rice fields.

A high SAR recorded for these soils (Table 1), ensues limitation of Ca^2+ ^and Mg^2+ ^due to Na^+^-induced displacement of these cations [42], which may be responsible for thin cyanobacterial population in these soils [39]. A relatively low concentration of Ca^2+ ^and very high Mg^2+ ^content of the soil from Jaddopur was due to the Mg^2+ ^induced deficiency of Ca^2+ ^[42]. In addition, the pH was found to range from neutral (Anei and Parsurampur, 7.40) to highly alkaline (Rauri, 9.04). A significant negative correlation (*P *< 0.05) observed between pH and the number of cyanobacteria in every soil sample, reflects the optimal pH for cyanobacterial growth at 7.5. This is supported by the fact that most diverse cyanobacterial group of this study, *Anabaena *and *Nostoc *prefer neutral to slightly alkaline soil [27]. Further, the concentration of available phosphorus in the soils varied between 13.65 (Chauki) and 103.71 ppm (Parsurampur). This fluctuation in the availability of phosphorus may also be due to the relative presence of monovalent (Na^+ ^and K^+^) and divalent (Ca^2+ ^and Mg^2+^) cations since the former are responsible for soluble and the latter for insoluble phosphorus. However, the regression analysis between available phosphorus and cyanobacterial populations does not demonstrate any significant relation. This can be due to the fact that 1.0 ppm available phosphorus has been reported to be sufficient for the growth of plants [43,44]. In contrast to this, available nitrogen was found to be negatively correlated (*P *< 0.05) with number of cyanobacteria (Figure 2B). This can be explained in the light of the observation of Fernández-Valiente et al. [45] who demonstrated inhibitory effect of nitrogen fertilizers on the growth of nitrogen-fixing cyanobacteria in paddy fields. Since the studied paddy fields have high diversity and population of nitrogen-fixing cyanobacteria, salinity-induced increase in available nitrogen [36] may eliminate their population. The positive correlation between available nitrogen with EC, Na^+ ^content, pH and SAR (*P *< 0.05) finds support with the observation of El-Karim et al. [36] that nitrogen availability in saline soil depends on EC, Na^+ ^and Ca^2+ ^content vis-a-vis SAR and pH.

**Table 1:** Physicochemical properties related to salinity level of the soils collected from respective sites.

S No	pH	EC (dS m^-1^)	Na^+ ^(ppm)	K^+ ^(ppm)	Na^+^/K^+^	SAR	Ca^2+ ^(ppm)	Mg^2+ ^(ppm)
1	8.53 ± 0.37	4.05 ± 0.28	2.91 ± 0.15	2.45 ± 0.16	1.18 ± 0.14	4.72 ± 0.55	0.49 ± 0.01	0.27 ± 0.01
2	8.76 ± 0.24	3.57 ± 0.21	7.48 ± 0.21	5.19 ± 0.23	1.44 ± 0.11	9.77 ± 0.82	0.79 ± 0.01	0.38 ± 0.02
3	8.12 ± 0.21	2.44 ± 0.10	2.93 ± 0.16	3.87 ± 0.11	0.75 ± 0.09	3.73 ± 0.21	0.69 ± 0.01	0.54 ± 0.01
4	7.93 ± 0.13	4.12 ± 0.23	3.31 ± 0.15	2.01 ± 0.09	1.64 ± 0.17	4.56 ± 0.11	0.72 ± 0.03	0.33 ± 0.02

5	8.68 ± 0.26	4.62 ± 0.28	2.37 ± 0.11	1.10 ± 0.05	2.15 ± 0.20	5.92 ± 0.21	0.17 ± 0.01	0.15 ± 0.01
6	7.83 ± 0.15	1.89 ± 0.11	2.80 ± 0.21	3.69 ± 0.12	0.75 ± 0.07	6.03 ± 0.42	0.32 ± 0.01	0.11 ± 0.01
7	7.76 ± 0.11	1.97 ± 0.24	2.80 ± 0.13	2.99 ± 0.15	0.93 ± 0.04	4.42 ± 0.20	0.29 ± 0.02	0.51 ± 0.02
8	7.45 ± 0.32	2.11 ± 0.13	3.07 ± 0.22	2.21 ± 0.16	1.38 ± 0.12	3.82 ± 0.19	0.66 ± 0.01	0.63 ± 0.02

9	8.81 ± 0.24	5.81 ± 0.14	7.92 ± 0.35	4.01 ± 0.24	1.97 ± 0.15	9.82 ± 0.64	0.83 ± 0.04	0.47 ± 0.01
10	7.95 ± 0.17	3.12 ± 0.12	3.42 ± 0.17	2.33 ± 0.11	1.46 ± 0.18	5.40 ± 0.32	0.47 ± 0.02	0.33 ± 0.01
11	8.69 ± 0.21	3.54 ± 0.13	5.58 ± 0.16	7.14 ± 0.38	0.78 ± 0.05	6.55 ± 0.21	1.13 ± 0.12	0.32 ± 0.01

12	8.19 ± 0.15	3.86 ± 0.18	2.98 ± 0.19	2.69 ± 0.23	1.10 ± 0.09	5.07 ± 0.47	0.54 ± 0.02	0.15 ± 0.01
13	7.40 ± 0.18	4.37 ± 0.16	2.12 ± 0.14	1.51 ± 0.05	1.40 ± 0.11	5.99 ± 0.38	0.14 ± 0.01	0.11 ± 0.01
14	8.54 ± 0.16	4.13 ± 0.20	3.37 ± 0.12	2.67 ± 0.17	1.26 ± 0.13	5.65 ± 0.32	0.62 ± 0.01	0.09 ± 0.005

15	8.06 ± 0.27	3.35 ± 0.11	4.47 ± 0.15	1.17 ± 0.11	3.82 ± 0.23	3.64 ± 0.12	0.65 ± 0.02	2.35 ± 0.09
16	9.04 ± 0.35	7.55 ± 0.27	9.15 ± 0.39	5.32 ± 0.21	1.71 ± 0.11	6.70 ± 0.52	2.03 ± 0.11	1.69 ± 0.05

17	7.40 ± 0.29	3.06 ± 0.21	2.13 ± 0.17	0.91 ± 0.01	2.34 ± 0.19	3.26 ± 0.11	0.60 ± 0.02	0.25 ± 0.01
18	8.06 ± 0.17	3.22 ± 0.10	3.11 ± 0.14	2.24 ± 0.23	1.38 ± 0.13	4.44 ± 0.31	0.81 ± 0.01	0.17 ± 0.01
19	8.62 ± 0.28	5.69 ± 0.17	4.16 ± 0.16	0.58 ± 0.01	7.17 ± 0.22	6.23 ± 0.24	0.83 ± 0.01	0.06 ± 0.001
20	8.39 ± 0.13	5.12 ± 0.22	5.61 ± 0.16	3.15 ± 0.12	1.78 ± 0.12	5.96 ± 0.36	1.58 ± 0.03	0.19 ± 0.01

The values are denoted as Mean ± SD.

EC, the most appropriate parameter to characterize soil salinity [46], was employed to classify the soil samples into two categories, normal (hereafter low) (< 4.0 ds m^-1^) and saline (hereafter high salinity) (> 4.0 ds m^-1^) soil [39]. This classification divided the sample soils into the following: (i) low salinity: Anei, Bardah, Bakesh, BHU, Jaddopur, Kataka, Madhopur, Maharupur, Makara, Misirpura, and Phootia, and (ii) high salinity: Aswania, Bithwal, Chauki, Kartihan, Parsurampur, Rajatalab, Rauri, Sewapuri and Teduababa. The regression analysis showed a significant negative correlation (*P *< 0.05) between the cyanobacterial population and EC (Figure 2A). Further, the influence of EC on cyanobacterial population was found highest among other parameters as reflected by a high *r *value (0.75) in regression analysis.

### Microscopic observation of cyanobacterial community

**Figure 3:**
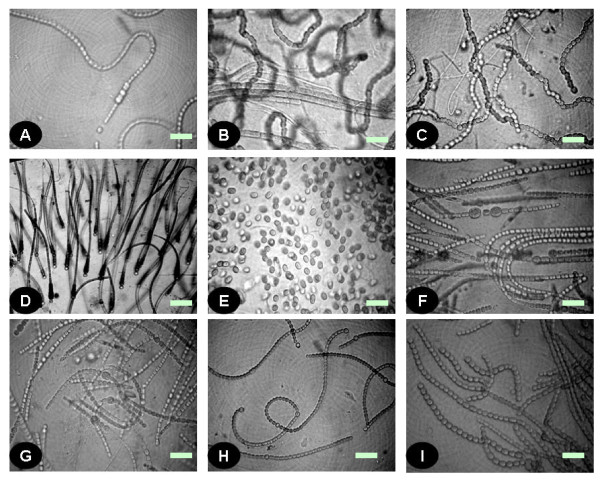
**Community of cyanobacteria collected from different rice fields as seen in microscope (resolution 40×)**. Some of the cyanobacterial genera that constituted the community were *Anabaena *(A, C and F), *Aulosira *(B), *Gloeotrichia *(D), *Aphanothece *(E), *Nostoc *(G and H) and *Hapalosiphon *(I). Bars, 10 μm.

Microscopic observation of the samples revealed the presence of diverse forms of cyanobacteria with most belonging to the order Nostocales. Cyanobacterial communities of rice fields were composed of the morphologically-defined genera *Anabaena, Nostoc, Aulosira, Cylindrospermum, Gloeotrichia, Rivularia *and *Tolypothrix *of the order Nostocales; *Oscillatoria, Lyngbya *and *Phormidium *of the Oscillatoriales; *Fischerella *and *Hapalosiphon *of Stigonematales; and *Aphanothece *and *Gloeothece *of the Chroococcales (Figure 3). Details of the microscopic analysis, in terms of cyanobacterial genera present at specific sites, are provided in Table 2. This observation is supported by the microscopic observations of Ali and Sandhu [31] and Tiwari and Singh [28], who reported occurrence of these cyanobacteria in saline soils of the Punjab, Pakistan and slightly acidic soil of rice fields of Manipur, India respectively. These cyanobacteria were previously characterized from different soil types in India [47]. Further, Pereira et al. [30] reported occurrence of different species of *Anabaena, Nostoc, Cylindrospermum *and *Gloeotrichia *in rice fields of Chile. In this study, the number of above-mentioned genera varied considerably across different samples. Samples from the hypersaline conditions also generally contained non-heterocystous filamentous and unicellular genera. In contrast to this, the mesosaline samples had a population with more heterocyst-forming cyanobacteria. The sample from BHU showed maximum cyanobacterial diversity with minimal diversity present at Kartihan and Rauri, in terms of cyanobacterial genera present. However, a general trend of a larger number of cyanobacteria in low salinity soils than high salinity soils was observed. In this study the presence of *Aulosira *in a large number of samples was supported by the results of Singh [2], who suggested it to be the dominant genus in Indian rice paddy fields.

**Table 2:** The name of experimental sites, their location, date of collection, the studied nutritional properties of the soil collected from respective sites and the microscopically observed cyanobacterial genera.

S no	District	Date of collection	Experimental sites	Available P (ppm × 10^-1^)	Available N (ppm × 10^-1^)	Microscopically observed cyanobacteria
1	Azamgarh	02.09.2006	Aswania	1.98 ± 0.25	1.97 ± 0.07	*Aulosira *sp., *Gloeotrichia *sp.* *Phormidium *sp.
2			Bardah	3.72 ± 0.06	3.08 ± 0.12	*Aulosira *sp., *Fischerella *sp., *Hapalosiphon *sp.,
3			Bakesh	3.02 ± 0.13	2.38 ± 0.09	*Anabaena *sp. (2 genera), *Aulosira *sp.
4			Chauki	1.37 ± 0.01	2.15 ± 0.10	*Anabaena *sp.*, *Nostoc *sp.

5	Chandauli	07.09.2006	Bithwal	3.38 ± 0.13	3.14 ± 0.13	*Aulosira *sp., *Nostoc *sp.
6			Madhopur	3.20 ± 0.15	1.47 ± 0.08	*Anabaena *sp., *Cylindrospermum *sp., *Nostoc *sp.
7			Misirpura	3.74 ± 0.37	1.22 ± 0.03	*Aulosira *sp.*, *Gloeotrichia *sp.
8			Phootia	2.59 ± 0.03	0.98 ± 0.02	*Anabaena *sp., *Cylindrospermum *sp.

9	Jaunpur	11.09.2006	Kartihan	5.89 ± 0.12	3.60 ± 0.12	*Gloeothece *sp.
10			Maharupur	6.73 ± 0.25	3.20 ± 0.15	*Anabaena *sp., *Phormidium *sp., *Rivularia *sp.
11			Makara	2.20 ± 0.17	1.13 ± 0.06	*Aulosira *sp., *Nostoc *sp. (2 genera*)

12	Mirzapur	15.09.2006	Kataka	2.16 ± 0.13	1.96 ± 0.05	*Aulosira *sp., *Hapalosiphon *sp., *Lyngbya *sp.
13			Parsurampur	10.37 ± 0.47	2.32 ± 0.12	*Anabaena *sp., *Aphanothece *sp.
14			Teduababa	4.63 ± 0.27	2.04 ± 0.13	*Aphanothece *sp.*, *Nostoc *sp., *Tolypothrix *sp.

15	SRD Nagar	22.09.2006	Jaddopur	3.23 ± 0.17	2.32 ± 0.09	*Nostoc *sp., *Hapalosiphon *sp.
16			Rauri	5.67 ± 0.69	3.78 ± 0.08	*Oscillatoria *sp.

17	Varanasi	27.09.2006	Anei	5.47 ± 0.37	1.76 ± 0.04	*Aulosira *sp., *Nostoc *sp., *Phormidium *sp.
18			BHU	4.59 ± 0.15	2.38 ± 0.14	*Aphanothece *sp., *Aulosira *sp., *Hapalosiphon *sp., *Lyngbya *sp., *Tolypothrix *sp.
19			Rajatalab	3.53 ± 0.11	3.18 ± 0.14	*Anabaena *sp.*, *Aulosira *sp., *Lyngbya *sp.
20			Sewapuri	3.22 ± 0.21	2.96 ± 0.08	*Fischerella *sp., *Hapalosiphon *sp.

Sign '*' denotes the genera not identified in molecular experiments.

### DGGE and molecular diversity

**Figure 4:**
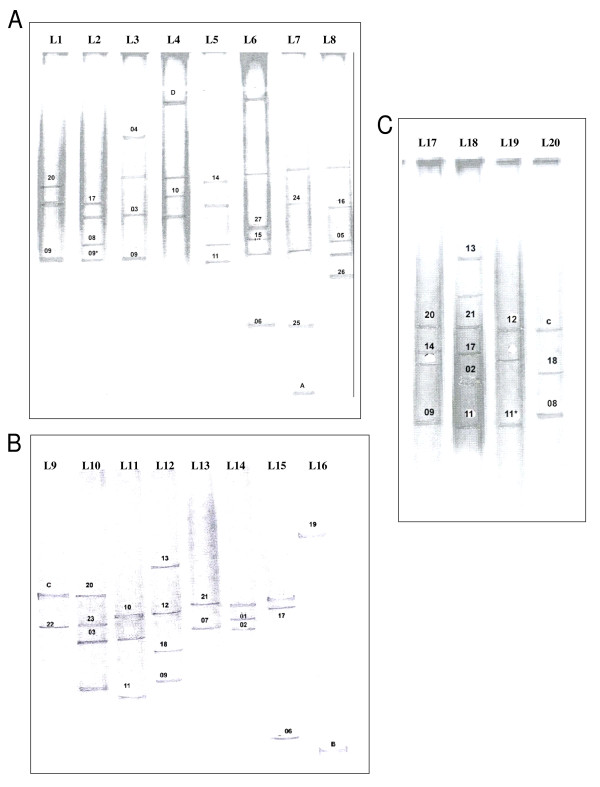
**DGGE band profile of the selected rice fields of (A) Azamgarh (1 – 4) and Chandauli (5 – 8), (B) Jaunpur (9 – 11), Mirzapur (12 – 14) and Sant Ravi Das Nagar (15 and 16), and (C) Varanasi (17 – 20)**. Numbered bands had similarity with the corresponding cyanobacteria in Table 3. Band marked "*" designate that the band present is at exact position on the gel as compared to corresponding band number. Only sections of the gels containing bands are shown. For details of the experimental sites see Table 2.

Molecular identification was used to support the morphological classification of cyanobacteria. This polyphasic approach to rice field cyanobacterial systematics provided a basis for comparison with previously identified taxa and also for future comparisons with taxa with similar physiologies. The characterization of cyanobacterial *16S rRNA *gene by PCR-DGGE is shown in Figure 4. Different banding patterns for each soil sample were observed, with a total of 73 delineated PCR products. Bands that showed significant reproducibility and minor changes in intensity after triplicate analyses were selected for further assessment. A total of 51 bands was selected for sequencing on the basis of their relative position on the gel and band intensity. However, BLAST analysis showed that only 31 DGGE bands had significant difference in their sequences. This may be due to that fact that DGGE is a very sensitive technique and can detect single nucleotide differences in a sequence [48], however, these differences may be insignificant in terms of percentage similarity across the entire molecule.

BLAST analysis revealed that 20 had close similarity to cultured cyanobacterial species, 7 with uncultured cyanobacterial species, 2 with uncultured and unclassified cyanobacteria, and 2 with plastids of diatoms and higher plants. Table 3 compares the data on the similarity of DGGE bands sequences to representatives from various cyanobacterial genera. Six PCR fragments (*A. doliolum, A. anomala, A. oryzae*, and *A. variabilis*) showed high similarity to *Anabaena *(96–99%), four (*N. endophytum, N. muscorum *and *Nostoc *sp.CCG3) with *Nostoc *(94–98%), two with *Aulosira *(*A. fertilissima *and *Aulosira *sp. PP615) (95% each.), *Cylindrospermum *(*Cylindrospermum *sp. A1345 and CENA33) (95 and 98%), *Gloeotrichia *(both with *G. echinulata*) (94 and 98%) and *Hapalosiphon *(*H. welwistchii *and *Hapalosiphon *sp. CCG6) (94 and 96%), and one each with *Rivularia *(*Rivularia *sp. PCC7116) (95%), *Tolypothrix *(*Tolypothrix *sp. PCC7415) (95%) and *Fischerella *(*F. muscicola*) (87%). Of the different *16S rRNA *gene sequences having similarity to non-heterocystous cyanobacteria, two had matches with *Lyngbya *(highest similarity with an uncultured *Lyngbya *sp. followed by *Phormidium corium *and *Microcoleus *sp.) (97 and 98% with uncultured *Lyngbya *sp.) and one each with *Oscillatoria *(*O. spongeliae*) (95% with uncultured *Oscillatoria *sp.), *Phormidium *(*P. inundatum*) (94%), *Aphanothece *(*Aphanothece *sp. OES3853) (96%) and *Gloeothece *(*Gloeothece *sp. SK40) (96% with uncultured *Gloeothece *sp.) (Table 3). The remaining two sequences showed similarity with unidentified cyanobacteria (at 95 and 96% identity), and one each with the plastids of a diatom (95%) and tobacco (96%). These cyanobacterial genera have also been characterized from rice fields of China and Thailand [34,49]. As reported elsewhere, most of the *16S rRNA *gene sequences obtained from DGGE did not share absolute identity to the sequences obtained from cultured cyanobacteria [50,51]. Of the sequences analyzed, 58% belonged to heterocystous Nostocalean genera having highest similarity with species of *Anabaena *and *Nostoc*. This finds support with the observation of Nayak and Prasanna [27]. Molecular data were found to agree with morphological attributes except in few cases, which may be due to either missing DGGE band information or phenotypic plasticity. Further, the results suggested a wide distribution of *Aulosira *across a range of salinities. In summary, the rice fields of Eastern Uttar Pradesh contained numerous species of the nitrogen-fixing *Anabaena *and *Nostoc *but have *Aulosira *(*A. fertilissima*) as the most cosmopolitan cyanobacterium.

**Table 3:** Selected DGGE bands showing similarity after sequencing and NCBI-BLAST search.

Band	No. of bases sequenced	Closed match		
		
		Description	GenBank accession number	% similarity
1	383	*Nostoc *sp. CCG3	DQ235803	94
2	388	*Tolypothrix *sp. PCC7415	AM230706	95
3	363	*Anabaena variabilis *NIES23	AF247593	98
4	394	*Anabaena doliolum *LCR1	EF066611	99
5	380	*Anabaena variabilis*	AB016520	97
6	384	*Nostoc endophytum *IAMM267	AB093490	98
7	349	*Anabaena *sp	X59559	98
8	364	*Fischerella muscicola *SAG1427	AB075985	87
9	400	*Aulosira *sp. PP615	AF527480	95
10	396	*Nostoc *sp.	Z82803	97
11	390	*Aulosira fertilissima *LCR4	EF066607	95
12	326	Uncultured *Lyngbya *sp. (*Phormidium corium*)	DQ146333 (EU068737)	97 (94)
13	358	Uncultured *Lyngbya *sp. (*Microcoleus *sp.)	DQ146331 (EF654075)	98 (95)
14	387	*Nostoc muscorum *CENA18	AY218827	94
15	392	*Anabaena anomala *LCR5	EF066608	96
16	354	*Anabaena oryzae *LCR2	EF066606	96
17	388	*Hapalosiphon *sp. CCG6	DQ235806	96
18	347	*Hapalosiphon welwistchii*	AY034793	94
19	378	Uncultured *Oscillatoria *sp. clone BME114 (*Oscillatoria spongeliae*)	DQ917838 (AF420445)	95 (89)
20	318	*Phormidium inundatum *SAG79.79	AM398801	94
21	317	*Aphanothece *sp. OES3853	DQ264198	96
22	330	Uncultured *Gloeothece *sp. (*Gloeothece *sp. SK40)	DQ072894 (AB067576)	96 (90)
23	357	*Rivularia *sp. PCC7116	AM230677	95
24	337	*Gloeotrichia echinulata *URA3	AM230705	94
25	376	*Gloeotrichia echinulata *URA3	AM230705	98
26	375	*Cylindrospermum *sp. A1345	DQ897365	98
27	352	*Cylindrospermum *sp. CENA33	AY218831	95
A	314	Tobacco Chloroplast	V00165	96
B	355	Uncultured cyanobacteria	AJ889114	96
C	364	Uncultured cyanobacteria	DQ514104	95
D	360	Uncultured diatom clone 100M1	DQ513978	95

The cyanobacterial strains mentioned in parenthesis represent the close similarity of respective DGGE bands with cultured cyanobacterial strains.

**Figure 5:**
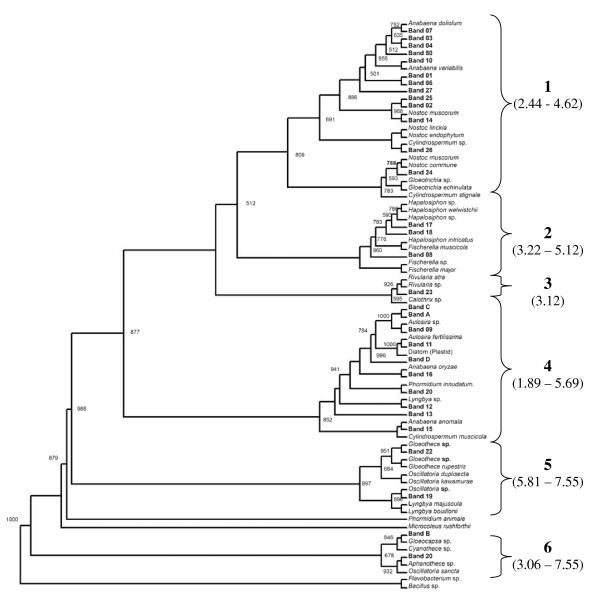
**Neighbor joining tree showing phylogenetic relationship of the sequenced DGGE bands**. Total 1000 bootstraps were performed and only more than 50% bootstrap support values are mentioned. All the phylogenetic analysis was performed using MEGA4 software. Numbers designate the clades. Values in parentheses indicate the range of soil salinities (in ds m^-1^) corresponding to the clade of cyanobacteria. For details of methodology refer materials and method section.

For assessment of the genetic relatedness among different cyanobacteria and description of the genetic diversity in relation to salinity levels of the different soils, a neighbor joining tree was constructed using additional 43 sequences of *16S rRNA *gene from cyanobacteria and plastids from database along with the 31 sequences obtained in this study (Figure 5). The *16S rRNA *gene sequences of *Bacillus *and *Flavobacterium *were used as out-groups. The phylogenetic tree showed six different clades supported by significant bootstrap values (1000 data resamplings), of which clade 1 belonged to the Nostocalean genera *Anabaena *and *Nostoc*. The correlation between the phylogenetic analysis and the distribution of cyanobacteria according to the salinity level may be particular for this ecosystem. Clade 1 also harbored two species of *Gloeotrichia *and one of *Cylindrospermum*. While all identified species of *Anabaena *were mainly confined to low salinity soils, except for *Anabaena *sp. (DGGE band 7) present in the sample from Parsurampur, all species of *Nostoc *were distributed over a range of salinities. Further, in this study, the two strains of *Cylindrospermum *and *Gloeotrichia *were found in soils with low salinity. The presence of *Nostoc *and *Anabaena *in same clade may be explained by the close genetic relatedness among these species [52]. Clade 2 exclusively contained branched heterocystous cyanobacteria of the genera *Hapalosiphon *and *Fischerella *from soils having a moderate to high salinity (Figure 5). The monophyletic origin of heterocystous taxa also finds support with the results of Gugger and Hoffmann [53] and Rajaniemi et al. [52]. This was accompanied by a small clade of Rivularia strains (3), obtained from soil of low salinity. Further, *Rivularia *and *Gloeotrichia*, both members of family Rivulariaceae were found in two different clades and are therefore genetically distant but morphologically similar [54].

*Aulosira, Phormidium *and *Lyngbya *formed clade 4 with sparse occurrence of *Anabaena oryzae*, *Anabaena anomala *and uncultured cyanobacteria (correspond to plastid *16S rRNA *gene). All of these species were found within sites having a wide range of salinities suggesting that the species in this clade (clade 4) are salt tolerant. Of these *Aulosira *emerged most widely distributed among the sample sites. Densely aggregated trichomes, macroscopic structure and the presence of a thick exopolysaccharide layer are the mechanisms that could permit this ecological adaptation [10]. Further, Zulpa de Caire et al. [55] have reported that salinity induces the synthesis of exopolysaccharides, which may help to tolerate high salinity. This molecular data highlights the role of *Aulosira *in the nitrogen budget of this region and in the potential reclamation of Usar (saline) land by aggregating the soil particles [2]. Sequences of clade 5 were present in high saline soil in this study and included *Oscillatoria *and *Gloeothece*. However, presence of cyanobacteria belonging to Chroococcales and Oscillatoriales in same clade may be due to their polyphyletic origin [56]. Rest of the species fell in clade 6 consisting of DGGE band similar to *Aphanothece *and uncultured cyanobacteria.

The nucleotide diversity was measured using Tajima-Nei model, which assumes equal substitution rates among character positions and between transitions and transversions. This model revealed the minimum evolutionary distance among members of Stigonematales. However, maximum genetic diversity was observed among the members of Nostocales. This may be due to the lower prevalence of Stigonematales compared to Nostocales in this phylogeny. Genetic distances were highest between Nostocales and Oscillatoriales and minimum between Chroococcales and Oscillatoriales.

Based on these observations, salinity tolerance in cyanobacteria would appear to be an adaptive trait that has evolved in parallel to speciation. Since this observation is based on *16S rRNA *gene, a highly conserved gene, the better picture of salinity tolerance would probably emerged using the gene sequences not much conserved so may represent the effect of environmental variables on diversity of cyanobacteria. This finds support with the work of Jaspers and Overmann [57] that microorganisms vary considerably in their genomes and thus ecophysiologies even with similar ribosomal gene sequences. Thus the mechanism for salinity tolerance may well be conserved in closely related cyanobacteria but differs considerably across this group of prokaryotes and may be attributed to genome plasticity in cyanobacteria.

These results demonstrated that the morphological characters and molecular phylogeny were almost congruent for these populations that contained a large number of filamentous and heterocystous species either with or without branching. It is established that the morphology of unicellular cyanobacteria is not as well defined and thus there is a considerable difference between morphology and the *16S rRNA *gene based phylogeny for this group. Therefore, the morphological characters for the identification of cyanobacteria and its agreement with the phylogenetic classification depends largely on the type of cyanobacteria in question.

### Salinity-induced changes in cyanobacterial community

**Figure 6:**
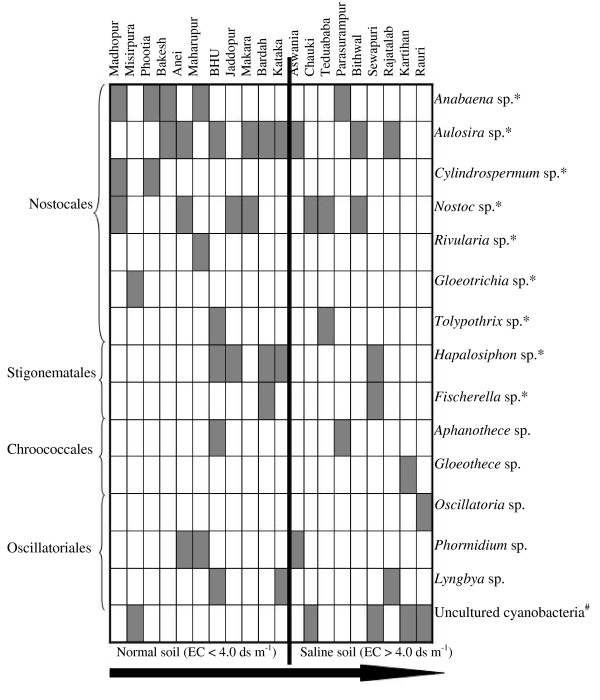
**Distribution of different cyanobacteria in the selected rice fields**. The arrow denotes the increasing level of salinity measured in terms of electrical conductivity. The cyanobacterial genus name includes all the DGGE bands showed similarity with the corresponding organism. Sings * and ^# ^represent the heterocystous cyanobacteria and unknown cyanobacteria respectively.

Figure 6 demonstrates the diversity of cyanobacteria in selected rice fields with different salinity levels. It was anticipated that salinity would play a dominant role in determining the cyanobacterial diversity in this environment. However, in some cases pH was also a determining factor. Low salinity soils had greater cyanobacterial diversity (as measured by *16S rRNA *gene DGGE bands) compared to soils with very high EC values. Dominance of cyanobacteria in these paddy fields based on band intensity on gels was not calculated, since gene abundance may be due to the varying number of *16S rRNA *gene per cell or PCR amplification biases [58]. However, the application of DGGE in combination with group-specific PCR for microbial ecology and diversity assessment is a widely accepted methodology [15]. In this study, *Nostoc *emerged as the second most prevalent and salinity adapted cyanobacterial genus after *Aulosira*. Again, this could be attributed to its colonial habit, macroscopic structures and thick extracellular mucilage, which provide protection from osmotic fluxes in the environment [59]. This observation finds support with the report of Mollenhauer et al. [60]. The presence of *Gloeothece *and *Oscillatoria *(*O. spongeliae*) in the rice fields having high salinity can be due to high nitrogen content in these soils and suggests that they can sustain high salt. In addition, species of *Aphanothece, Hapalosiphon *and *Rivularia *present in the soil of Jaddopur may uphold high Mg^2+ ^in comparison to the cyanobacteria from other regions. High concentrations of Ca^2+ ^and low Mg^2+ ^in the soils of Rauri and Rajatalab, respectively, also seemed to select for a specific cyanobacterial community. Finally, the most diverse population of cyanobacteria (as revealed by DGGE) was from the rice fields of site with low salinity that was characterized as having neutral to slightly alkaline pH and low EC while being phosphorus replete.

Relatively low salinity levels favored the growth of heterocystous cyanobacteria while high salinity (more than 4 ds m^-1^) appeared to select for non-heterocystous species (Figure 6). Since high salinity reduces ammonia volatilization [36], the high nitrogen content in saline soil would be detrimental for heterocystous cyanobacteria [45]. Likewise, Staal et al. [61] demonstrated that in less saline conditions, the glycolipid envelop of a heterocyst provides a selective advantage over non-heterocystous cyanobacteria. The distribution of phylogenetic relationships across environmental gradients is not well understood. However, here we obtained a distinct relationship between cyanobacterial occurrence and salinity levels using both morphological and molecular data. This ecosystem is characterized by numerous overlapping factors other than salinity. Therefore, the community structure that was described here may vary with other environmental perturbations.

Soil salinity is one of the major determinants of cyanobacterial distribution and diversity in the rice fields of Eastern Uttar Pradesh. This study has shown that salinity influences cyanobacterial species distribution in rice fields, whereby high salinity soils selectively support the growth of non-heterocystous cyanobacterial populations. Threats imposed by ever-increasing salinity have resulted in thin cyanobacterial populations that lead to a reduction in biological nitrogen fixation and increased demand of chemical fertilizers in the paddy fields.

## Methods

### Sampling sites, sampling and biochemicals

**Figure 7:**
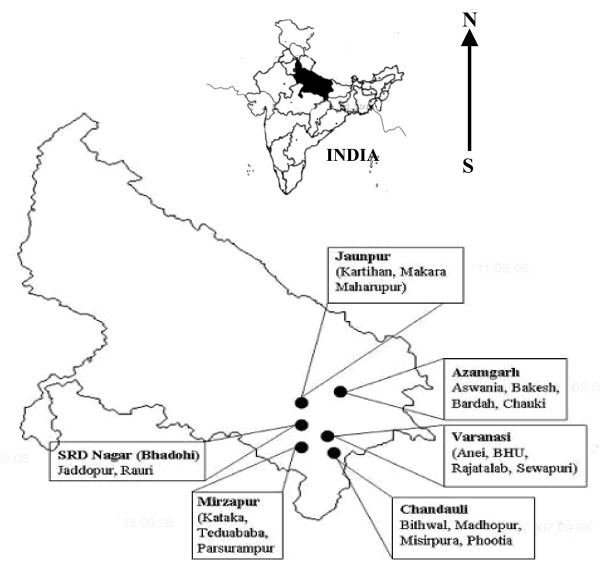
**Map of experimental site**. The map of India showing location of Uttar Pradesh and map of Uttar Pradesh (23°52' N and 31°28' N latitude and 77°3' E and 84°39' E longitude) showing the experimental sites in six districts (the location of each district is given in the material and methods section).

A total of 20 rice fields situated in six districts of Eastern Uttar Pradesh were selected for sampling (situated from 24°56' to 26°06' N and 81°14' to 83°19' E). The names of villages and dates of sampling are listed in Figure 7 and translated into sample numbers (see Table 2). The sites were selected having consideration that (i) the sampling should be random, and (ii) rice fields should not be much disturbed. The samples were collected during September 2006, which is the monsoon season in this part of India and considered the optimal period for cyanobacterial growth. Climatic factors such as light, humidity and rainfall were uniform since all sites are located in the same geographical region. Although, some reports state that cyanobacterial diversity is better correlated with water characteristics in rice fields [62], others describe an affiliation with soil properties [63]. In view of the presence of cyanobacteria deep within the soil of rice fields [34], latter proposal seems more feasible. The soil samples were collected on sunny days having temperatures between 27 to 30°C and between the hours of 10.00 and 11.00 AM (IST). The collected samples were transported to the laboratory on ice and stored at -20°C. All experiments relating to the physicochemical properties of soil and PCR-DGGE were performed in triplicate and repeated at least twice to ensure the reproducibility of the results. All biochemicals were procured from the Sigma Chemical Co. USA unless otherwise specified.

### Chemical composition of soil samples

For the analysis of pH and electrical conductivity (EC), 50 ml of double-distilled deionized Milli-Q water was added to 10 g of soil and homogenized. The suspension was subjected to centrifugation at 10,000 *g *for 10 min. The supernatant was used for the measurement of pH and EC using a pH (Systronics, India) and EC (Hanna Instruments, Portugal) meters respectively.

For Na^+^, K^+^, Ca^2+ ^and Mg^2+ ^analysis, 5 g of soil was pre-digested with concentrated H_2_SO_4_. To this, H_2_O_2 _was added drop-wise until the solution became colorless. This solution was incubated on a hot plate at 70°C for 2 h and the process was repeated thrice. When the solution became dry, 10 ml of double-distilled deionized Milli-Q water was added. The resulting solution was analyzed using a Perkin Elmer 2380 atomic absorption spectrophotometer [64]. The sodium absorption ratio (SAR) was calculated using the formula:

Available phosphorus was measured using the method of Olsen et al. [65]. One gram of soil was mixed with 20 ml of 0.5 M NaHCO_3 _(pH 8.5) and 200 mg activated charcoal. This was shaken for 30 min at 200 *g *in an environmental shaker (Model-3597-ICOGMPR, USA) maintained at 25°C followed by filtration through Whatmann No. 1 filter paper. The pH of extract was maintained to 5.0 using concentrated H_2_SO_4_. The extract was then quantified for phosphorus content using molybdophosphoric acid [66].

Ammonia- and nitrate-nitrogen were measured by extracting 10 g of soil in 50 ml 2 M KCl and Morgan's Reagent (pH 4.8) respectively. In each sample 250 mg activated charcoal was added to obtain the clear supernatant. These were subjected to filtration through Whatmann No. 1 filter paper and used for ammonia-nitrogen measurement by phenate method [67] and nitrate-nitrogen estimation using the procedure described by Jackson [68]. Data were presented in terms of total nitrogen (combination of ammonia- and nitrate-nitrogen).

### Microscopic observation

The samples were microscopically analyzed using a trinocular microscope (Kyowa, Getner, Japan). The morphological characteristics of the cyanobacteria were compared with those in the literature of Desikachary [3] and Geitler [12]. Photo-documentation was performed with a digital camera and 40× magnification (Olympus).

### Genomic DNA isolation and PCR amplification of 16S rRNA gene

Total genomic DNA from the natural samples (paddy field soil and cyanobacterial mat) was isolated using the phenol and lysozyme-free method of Srivastava et al. [69]. The DNA thus obtained was passed through a spin column containing Sepharose 4B for the removal of salts and humic acids. Soil and mat samples were selected for DNA isolation from perennating bodies and growing cyanobacteria respectively. The primers CYA106F (CGC ACG GGT GAG TAA CGC GTG A) and CYA359F (GGG GAA TYT TCC GCA ATG GG) with a 40 nucleotide GC clamp (5'-CGC CCG CCG CGC CCC GCG CCG GTC CCG CCG CCC CCG CCC G-3') on the 5' end (forward primer) and CYA781R (equimolar mixture of CYA781Ra (GAC TAC TGG GGT ATC TAA TCC CAT T) and CYA781Rb (GAC TAC AGG GGT ATC TAA TCC CTT T)) (reverse primers) for amplification of a segment of cyanobacterial *16S rRNA *gene [70] were synthesized (Sigma Chemical Co., USA). A semi-nested PCR reaction was carried out with the first reaction using primers CYA106F and CYA781R followed by a reaction with primers CYA359F and CYA781R. PCR was performed in a 25 μl final volume of reaction mixture containing 100 ng of DNA, 2.5 μl of 10× PCR buffer with 15 mM MgCl_2_, 200 μM dNTPs, 10 pmol of each primer, 200 μg bovine serum albumin (nuclease free) and 0.2 U *Taq *DNA polymerase (Bangalore Genei, India) in an Icycler (Bio-Rad, USA). The thermal cycling profile was as follows: initial denaturation for 3 min at 94°C, followed by 35 amplification cycles each consisting of 1.5 min denaturation at 94°C, 1 min annealing at 59°C, and a 2 min elongation at 72°C, with a final 5 min elongation at 72°C.

### DGGE analysis

The PCR products of mat and soil samples obtained after the second PCR reaction were subjected to DGGE analysis using the DGGE-2001 system (C.B.S. Scientific Company, Inc. USA). An aliquot of 25 μl of PCR product was mixed with 5 μl of 10× gel loading solution (100% glycerol, 0.25% bromophenol blue and 0.25% xylenecyanole) and applied directly onto a 6% polyacryamide gel (acrylamide/bis 38.93/1.07) (w/v) in 1× Tris-acetate-EDTA (TAE) buffer with a linear 35–55% denaturant gradient (100% denaturant solution contained 7 M urea and 40% (v/v) deionized formamide). A gradient dye solution (0.5% bromophenol blue, 0.5% xylenecyanole and 1× TAE buffer) was used to check the gradient formation. DGGE was carried out at 60°C (constant temperature) for 16 h at 100 V (35 mA). The gel was stained for 15 min with ethidium bromide (1 μg ml^-1 ^in 1× TAE buffer) and visualized by UV transillumination and photographed. The PCR (*16S rRNA *gene) products of cultured *Anabaena *PCC7120, *Anabaena doliolum *LCR1 and *Hapalosiphon intricatus *BHULCR1 were included as genetic markers on each gel alongside the environmental samples (data not shown).

### Sequencing of 16S rRNA gene

A total of 51 bands were carefully excised from the DGGE gels using an autoclaved surgical scalpel and re-suspended in sterile Milli-Q water for 3 h to elute DNA from the gel matrix [71]. The eluted PCR products were used as template for re-amplification of the corresponding DGGE bands using the primer set CYA359F (with GC clamps) and CYA781R, and subsequently followed by another DGGE as described above. Only reactions that resulted in a single band with the predicted mobility were processed further. The specific bands were again excised and re-amplified. PCR conditions were the same as mentioned above for *16S rRNA *gene amplification except the primers did not have the GC clamp and 0.5 μl template DNA was used. PCR products were sequenced commercially (Bangalore Genei, India) with the same amplification primers in separate reactions. However, only 31 DGGE bands which showed significant difference in their sequences were selected for further analysis.

### Phylogenetic analyses

A multiple alignment was produced using the CLUSTAL_X ver. 2 [72] and manually corrected using JalView. Bands with identical mobility on DGGE gel were considered to have identical sequences. Sequence similarity between the 31 different partial *16S rRNA *gene sequences resulting from DGGE analysis were deposited in GenBank and assessed by BLASTN [73] homology searches using the nonredundant NCBI GenBank database. In addition to this, 43 *16S rRNA *gene sequences from GenBank, which showed the closest similarity with the different DGGE-PCR products, were also included in the multiple alignment. Pair-wise distance matrices were calculated using the Tajima-Nei method [74]. Character positions with gaps were deleted. The *16S rRNA *gene sequences of cyanobacteria were classified into phylogenetic groups as proposed by Desikachary [3] for the determination of genetic variability within and between the groups. Phylogenetic trees were constructed using the neighbor-joining algorithm [75] provided in MEGA4 [76]. One thousand bootstrap replicates of the alignment data were also performed and the consensus tree was constructed.

### Data analyses

Results of the soil analysis were statistically analyzed using one-way ANOVA followed by correlation coefficient (*r*) analysis using SPSS 10.0. Principal component analysis was performed using Statistica 8.0. For PCA analysis, the soil analysis data presented in Tables 1 and 2 were considered. The clustering in the PCA was performed as per Coeyne et al. [77] using cluster analysis (similarity measure: Pearson or product-moment correlation coefficient; clustering method: UPGMA). Further, to correlate cyanobacterial abundance and the physicochemical properties of soil (EC and available nitrogen), each band on a DGGE gel was treated as an individual species. The total number of bands present in any individual lane was considered to be the cyanobacterial diversity in that soil sample. Further, for accurate estimation of diversity, microscopic observations were also compared with the molecular data. Three independent variables were used for each experiment.

### Nucleotide accession numbers

The 31 partial *16S rRNA *gene sequences which showed significant difference in their sequence were analyzed and taxonomically assigned using the BLAST program of NCBI. The sequences were deposited in the database under the accession numbers [GenBank: EF619446] to [GenBank: EF619472] and [GenBank: EF624387] to [GenBank: EF624390].

## Competing interests

The authors declare that they have no competing interests.

## Authors' contributions

AKS designed and conducted the experiments in consultation with LCR, analyzed the data and drafted the manuscript. PB helped with the DGGE, phylogenetic analysis and drafting of the manuscript. AK carried out the phosphate and nitrogen measurement experiments. BAN assisted with the phylogenetic analysis, data interpretation and drafting the manuscript.

## Authors' information

AKS is Assistant Professor in Mizoram University, India. LCR and BAN are Professors in Banaras Hindu University, India and University of New South Wales, Australia, respectively.
